# Illuminating the Signals Job Seekers Receive from an Employer's Community Involvement and Environmental Sustainability Practices: Insights into Why Most Job Seekers Are Attracted, Others Are Indifferent, and a Few Are Repelled

**DOI:** 10.3389/fpsyg.2016.00426

**Published:** 2016-03-31

**Authors:** David A. Jones, Chelsea R. Willness, Kristin W. Heller

**Affiliations:** ^1^Grossman School of Business, The University of VermontBurlington, VT, USA; ^2^Human Resources and Organizational Behaviour, Edwards School of Business, University of SaskatchewanSaskatoon, SK, Canada

**Keywords:** corporate social responsibility, signaling theory, sustainable business, community involvement, employee volunteering, employee recruitment, skepticism, cynicism

## Abstract

Evidence shows that job seekers tend to be attracted to employers known for their corporate social responsibility (CSR), but relatively little is known about the underlying psychological processes. Moreover, the literature is silent about whether and why some job seekers are unaffected, or even repelled by, an employer's CSR. We conducted a substantive replication of recent empirical support for three signal-based mechanisms by adapting the experimental manipulation used in a prior study while employing an alternative approach to analyzing a distinctly different type of data. We also extended prior work by examining other possible explanatory mechanisms and exploring potentially negative reactions to CSR. Using signaling theory as an overarching framework, we assessed research questions and tested hypotheses grounded in theories of employee recruitment and the psychology of CSR, specifying how an employer's CSR practices send signals from which job seekers draw inferences about unknown working conditions, thereby affecting their attraction to the employer. Study participants (*N* = 108) reviewed the webpages of two hiring companies and responded to open-ended questions about each employer. We content-analyzed written responses pertaining to one employer's webpages in which we embedded an experimental manipulation of information about the employer's community involvement or its environmentally sustainable practices. The results supported hypotheses that corroborate prior evidence for the “perceived value fit” and “expected employee treatment” mechanisms, and provided some, but relatively limited, support for the “anticipated pride” mechanism. Assessment of research questions highlighted previously undiscovered signal-based mechanisms that might help explain job seekers' attraction to CSR (e.g., inferences about the employer's positive work environment and financial standing, and the nature of its employees). Results also showed that a few people were *less* attracted because of the employer's CSR practices. Analyses among those individuals, combined with one-third of the sample who reported their attraction was unaffected by the employer's CSR, provided insights about when and why CSR fails to enhance attraction, such as when job seekers focus on other priorities, or are deeply skeptical and cynical about the employer's CSR. We discuss the implications for advancing a signal-based theory of CSR and employee recruitment, and recruitment practice.

## Introduction

Research on employee recruitment has highlighted several factors that influence the extent to which job seekers are attracted to working for a given employer (Breaugh, 2008; Uggerslev et al., 2012). Among these factors, an employer's overall image and reputation is known to affect recruitment outcomes, and its image pertaining to corporate social responsibility (CSR) has emerged as an important factor that can shape the employer's attractiveness to job seekers (Jones and Willness, 2013). CSR refers to an organization's “actions and policies that take into account stakeholders” expectations and the triple bottom line of economic, social, and environmental performance (Aguinis, 2011, p. 855). In this study we focus on two specific forms of CSR that reflect an organization's discretionary external CSR actions pertaining to community involvement and environmentally sustainable practices. Community involvement includes philanthropy and support for employee volunteerism (e.g., Jones, 2010; Grant, 2012). Environmentally sustainable practices include policies that encourage employees to conserve energy and resources, efforts to improve the environmental impact of the supply chain, and programs to encourage environmental awareness (e.g., Christmann, 2000).

Studies suggest that job seekers tend to view organizations with strong CSR practices as more attractive employment options (Bauer and Aiman-Smith, 1996; Turban and Greening, 1997; Greening and Turban, 2000; Aiman-Smith et al., 2001; Backhaus et al., 2002; Behrend et al., 2009; Tsai and Yang, 2010; Kim and Parke, 2011; Jones et al., 2014). CSR, then, can offer employers a source of competitive advantage: Studies show that by drawing more applicants, companies substantially increase their chance of hiring top performers (Ployhart, 2006; Breaugh, 2008).

Until recently, however, the underlying psychological processes that explain the effects of CSR on recruitment outcomes were poorly understood. Findings reported by Jones et al. (2014) across two studies—a controlled experiment and a field study—provide evidence for three underlying signal-based mechanisms that help explain *why* job seekers tend to be attracted by CSR. We conducted a substantive replication and tested hypotheses about these three mechanisms by adapting the experimental manipulation and study stimuli from Jones et al. (2014, Study 1) while employing an alternative approach to collecting and analyzing data that allowed study participants to *tell us* what they inferred from an employer's CSR, rather than *react to* measures of what we think they might infer from CSR. Our methodology allowed us to collect data that were untainted by exposing participants to survey items and measures of underlying mechanisms that might prime them to respond in ways that are artificially more consistent with their attraction to an employer known for its CSR practices.

We also designed our study to extend prior findings and advance this literature in several ways. We assessed two research questions to uncover other potential mechanisms and explore possible differences between two types of CSR practices, and ultimately contribute to the understanding of job seekers' attraction to employers known for their CSR. We also sought to inform theory and future research by addressing a major gap in the psychology of job seeker responses to CSR. Extant research is virtually silent about whether and why some job seekers are unaffected by an employer's CSR, and even repelled by it. Accordingly, we examined a third research question about whether and why some job seekers are less attracted to an employer because of its CSR.

## Theory and hypotheses

### Job seeker attraction to an employer's CSR: three signal-based mechanisms

Job seekers are motivated to understand what it's like to work for a potential employer, but they usually lack the information to do so. Signaling theory suggests job seekers use whatever information they have as signals from which they make inferences that inform their employment decisions (Rynes, 1991). Scholars have suggested that signaling theory is well-suited for understanding job seekers' responses to CSR, as well as other recruitment attitudes and behaviors (Jones and Willness, 2013), but in the broader recruitment literature signal-based mechanisms are typically not well-specified conceptually, much less directly tested (Celani and Singh, 2010).

In one exception, Jones et al. (2014) derived hypotheses from signaling theory about three signal-based mechanisms that were tested together in the same models across two studies. These authors focused on the effects of an employer's community involvement and environmentally sustainable practices—the same two types of CSR practices on which we focused in the present study. Jones et al. found support for each of the three signal-based mechanisms, which we describe in greater detail in the sections below: CSR practices send signals about the organization's values, reputation and prestige, and pro-social orientation, which respectively inform job seekers' perceived value fit with the organization, anticipated pride as an employee, and expected treatment as an employee.

In Study 1, Jones et al. (2014) created a simulated job search context and used an experimental design in which they embedded CSR manipulations within the webpages of a fictitious employer. The authors tested whether the effects of the CSR manipulations on participants' attraction to a target employer were mediated by measures representing each of the three signal-based mechanisms. They found support for the effects of both types of CSR practices on attraction through two mechanisms when all three were tested together, and support for the third mechanism when one other particularly strong mechanism was removed from the model. In their Study 2, Jones et al. again tested mediated effects through the three signal-based mechanisms entered together in the same models, except this time using field data collected from actual job seekers. Study 2 participants were job seekers attending a job fair who completed a survey about one of the hiring companies at the job fair that they identified as one in which they were particularly interested. The authors operationalized CSR practices in two ways: job seekers' perceptions of the extent of the organization's CSR practices, and an independent measure of CSR practices based on coding the recruitment materials used by each employer present at the job fair. Across both operationalizations of CSR, they found support for mediated effects of community involvement practices through each of the three mediating mechanisms on job seekers' attraction to the hiring organization. Next, we present the rationale for our three study hypotheses after a more detailed description of each of the three signal-based mechanisms.

### Signals about an employer's values that inform job seekers' perceived value fit

At the broadest level, person-organization fit pertains to the perceived compatibility between an employee and an organization (Kristof, 1996), and fit includes the congruence between the two parties' values (Chatman, 1991). Meta-analytic evidence shows that person-organization fit is among the strongest predictors of recruiting outcomes (Chapman et al., 2005). Recruitment researchers have recognized that an organization's CSR practices send signals about its values, which likely increases its attractiveness when people perceive that these organizational values are a good fit with their own values (Turban and Greening, 1997; Greening and Turban, 2000; Aiman-Smith et al., 2001; Backhaus et al., 2002; Behrend et al., 2009). Thus, an employer's CSR actions send signals to job seekers about its *organizational values*, like being committed to the community or caring about reducing its negative impact on the natural environment. Signals from CSR about such values link CSR to organizational attractiveness via a signal-based mechanism of *perceived value fit*.

Jones et al. (2014) found support for this explanatory mechanism, which they tested in multiple ways across two studies. In their experimental design used in Study 1, they tested whether the effects of the CSR manipulations on job seekers' attraction in a simulated job search context was mediated by a direct measure of perceived value fit (Kristof, 1996). Also in Study 1, they tested an alternative operationalization of value fit using measures of individual differences pertinent to community involvement and environmentally sustainable practices. Both sets of analyses provided support for the perceived value-fit mechanism. In Study 2, their analyses of field data showed that job seekers' perceptions of community involvement, and separate analyses of independent ratings of community involvement based on the employers' recruitment materials, had indirect effects on job seeker attraction through perceived value fit. In both Studies 1 and 2, support for the perceived value fit mechanism was found while controlling for the effects through the two other hypothesized signal-based mechanisms that we soon describe. Other experimental studies on employee recruitment have found support for perceived fit when tested on its own (Kim and Parke, 2011; Gully et al., 2013).

*Hypothesis 1: An employer's community involvement and environmentally sustainable practices send signals to job seekers about its organizational values from which job seekers infer perceived value fit (how well the employer's values fit with their own values)*.

### Signals about an employer's prestige that inform job seekers' anticipated pride

An organization's reputation is affected by its CSR (Fombrun and Shanley, 1990), and reputation sends signals that can influence job seekers' perceptions about a potential employer (Cable and Turban, 2003). When an employer is known for its CSR practices, researchers have argued, it signals to job seekers that the organization is prestigious and well-regarded by others (Behrend et al., 2009). This signal, in turn, informs feelings of pride that job seekers anticipate experiencing if they were associated with the organization as one of its employees (Jones and Willness, 2013).

This signal-based mechanism of *anticipated pride*, which follows from a signal from CSR about the *organization's prestige*, is rooted in principles of social identity theory (Ashforth and Mael, 1989; Tajfel and Turner, 1992; Collins and Han, 2004). Individuals derive aspects of their identities through their affiliations with social groups, including the organization in which they are employed (e.g., Dutton and Dukerich, 1991), particularly when identifying with their employer enhances their self-worth (Ashforth et al., 2008). Connecting organizational prestige to anticipated pride, scholars have noted that people “feel proud of being part of a well-respected organization, as it strengthens their feelings of self-worth to bask in reflected glory” (Smidts et al., 2001, p. 1051). Thus, an organization with a strong reputation for CSR would be viewed as prestigious, and the feelings of pride that job seekers anticipate experiencing make the organization more attractive as a potential employer.

In their Study 1, Jones et al. (2014) tested whether anticipated pride mediates the effects of community involvement and environmentally sustainable practices on organizational attractiveness, and they found support for these hypothesized effects above and beyond the two other signal-based mechanisms. In Study 2, they tested the mediating role of organizational prestige (the proposed signal), rather than anticipated pride (the proposed signal-based mechanism), which was supported for the effects of the employer's community involvement. Behrend et al. (2009) likewise found support for a similar signal effect from organizational prestige, although they tested it in isolation (i.e., not in the context of other signal-based mechanisms).

*Hypothesis 2: An employer's community involvement and environmentally sustainable practices send signals to job seekers about the employer's reputation and prestige from which they infer how proud they would feel as one of its employees*.

### Signals about the employer's prosocial orientation that inform job seekers' expected treatment

Theory on the psychology of CSR suggests that employees view externally-directed CSR activities (e.g., community involvement and environmentally sustainable practices) as evidence that an organization is concerned about the just treatment of others (Aguilera et al., 2007). Thus, CSR signals to job seekers that the organization has a *prosocial orientation*: a sincere concern for the well-being of others (Grant et al., 2008). This signal subsequently informs a signal-based mechanism about *expected treatment*; that is, people's perceptions about how well the organization treats its employees, given its care for people in general. For job seekers, by extension, such signals inform their perceptions about how favorably they would be treated if they were to work for that organization. In turn, job seekers' expectations about being treated with dignity, respect and fairness, for example, ultimately affects their attraction.

Rupp and colleagues found that an employer's community involvement and environmental sustainability interacted with information about employee treatment to predict participants' attraction to a fictitious employer (Rupp et al., 2013). To our knowledge, however, the expected treatment mechanism has only been tested directly by Jones et al. (2014), who found support for this mechanism in their field study with respect to community involvement, and in their experimental study for both community involvement and environmentally sustainable practices after removing a particularly strong effect through anticipated pride from the model.

*Hypothesis 3: An employer's community involvement and environmentally sustainable practices send signals to job seekers about the employer's prosocial orientation from which they infer how well they expect to be treated as one of its employees*.

### Other reasons job seekers are attracted: unexplored signals sent by CSR

Researchers have speculated about other potential signals from CSR that might affect inferences that job seekers draw that ultimately influence their attraction to the employer. An employer's CSR may signal that it can afford to invest in discretionary environmental and social practices, from which job seekers may infer that the organization is financially stable, has good future growth prospects, or pays above average wages (Jones and Willness, 2013). Other signals might be about the type of people who work in the organization (Jones et al., 2014) or the overall work climate (Zhang and Gowan, 2012). We sought to extend Jones et al. (2014) by investigating these and other potentially relevant signals from CSR.

Research Question 1: Does an employer's community involvement and environmentally sustainable practices send other, previously unstudied, signals to job seekers that might plausibly affect their attraction to an employer?

### Differences between community involvement and environmental sustainability

Researchers have suggested that the nature of CSR practices may influence the relative strength of their effects through different signal-based mechanisms (Jones and Willness, 2013). Based on their results, Jones et al. (2014) speculated that an employer's community involvement may have relatively stronger effects through the anticipated pride mechanism, as such practices may be viewed as more discretionary and less directly linked to the “bottom line” compared to pro-environmental practices that often produce meaningful cost savings, thereby rendering community involvement more commendable and prestige-worthy. These authors also suggested that the expected treatment mechanism may be stronger for an employer's community involvement because it sends signals about the employer's prosocial orientation based on its treatment of people in the community, which logically extends to its own people (i.e., employees) more so than would signals based on its treatment of the natural environment.

Research Question 2: Do the signals and associated inferences job seekers draw differ based on information about an employer's community involvement vs. its environmentally sustainable practices?

### Potential negative reactions to CSR practices

Researchers have urged scholars to explore whether some job seekers react *negatively* to an employer's CSR practices (Willness and Jones, 2013), however the literature is all but silent as to whether and why some job seekers are unaffected or even repelled by an employer's CSR. Willness and Jones (2013) asserted that negative reactions may occur when job seekers question the credibility of CSR claims (i.e., greenwashing) or the benevolent nature of the employer's underlying motives (i.e., attributing purely self-interested motives for its CSR practices). These authors suggested that skepticism and negative reactions are more likely to occur when job seekers learn about the employer's CSR through media it owns and controls, such as press releases from its public relations department. Extending this logic to corporate websites, some job seekers may be skeptical of CSR information presented on an employer's webpages because the company created those messages (compared to, for example, learning about its CSR through its inclusion on a third party's list of top corporate citizens). Thus, some job seekers might discount or be repelled by an employer's descriptions of its CSR when they view its practices or the employer's underlying motives with suspicion and skepticism. Also plausible is that some job seekers are repelled by CSR because they hold values in opposition to any attempts by for-profit companies to address societal ills or environmental challenges.

Research Question 3: Are some job seekers less attracted to an employer because of its community involvement and environmentally sustainable practices, and, if so, why might this occur?

## Materials and methods

### Participants

Study participants were 108 undergraduate students who were enrolled in one of three business courses at a university in the Northeastern United States. The participants included 47 females and 61 males, most of whom were in their second (32.41%), third (45.37%), or fourth (20.37%) year of their undergraduate degree program. On average, they were 20.57 years of age (*SD* = 1.36) and had 4.05 years of work experience (*SD* = 2.40). Slightly more than half (*n* = 59, 54.63%) indicated they were looking for a new employment position at the time of their participation, and three-quarters indicated they intended to seek a new employment position within the next 6 months (*n* = 81, 75.00%). Among the 56 participants who were employed at the time of the study (51.85%), their average tenure with their employer was 1.68 years (*SD* = 1.59) and they worked 18.49 h (*SD* = 11.51) per week.

### Study procedure, employer web pages, and experimental manipulations

The three course professors invited students to participate in the study in return for a 1% bonus applied toward their final course grade, and they instructed interested students to email one of the authors. We randomly assigned interested students to an experimental condition and provided a link to the corresponding version of the confidential online survey. Upon accessing the survey, participants were informed about what participating in the study would entail, and that it would require about 30 min of their time. After they provided demographic information, we asked participants to create a unique study ID number that they could then give to their professors so they could allocate the bonus credit after verifying the validity of the students' participation using a list of ID numbers we provided. This process allowed us to maintain anonymity by avoiding the collection of participants' names or other identifying information.

Participants were instructed to review the webpages of two fictitious apparel companies that were hiring in the local area. The webpages were modeled after those used in Study 1 of Jones et al. (2014). The pages were designed to be realistic, and were formatted in a professional manner, including pictures, logos, links to other pages, and the type of information that would be found on a real employer's webpages. A similar amount of information was presented on each company's webpages. Three pages were presented for each employer that comprised general information about the organization, such as the company's history, its business principles, and its locations that included operations in the area in which study participants lived. Each employer's webpages also included a fourth page where several available job postings were advertised in a variety of functional roles relevant to the business student participants, including retail sales, marketing, product design, and human resources. We asked participants to “pretend as though you are currently seeking a job” and to “act as though you are interested in the kinds of employment positions they have open.”

Participants first reviewed the webpages for a company called Cotton One, which we included only to deflect attention from the main focus of the study and to create a more realistic simulation of a job seeker's consideration of multiple potential employment options. Our analyses focused on participants' reactions to the webpages of a second employer called “Active Style.” This target employer's webpages included a fifth webpage titled “We Care.” On this page we embedded a two-level experimental manipulation: the presence of information about the employer's Community Involvement (CI condition; *n* = 54) or business practices that are considered to reflect Environmental Sustainability (ES condition; *n* = 54). We used the same tone, layout, and wording for the “We Care” webpages in the CI and ES conditions (see the Appendix). For the CI condition, the “We Care” page contained information regarding Active Style's philanthropy and volunteerism program; for the ES condition, the “We Care” page contained information about their ecological philanthropy and a recycling program.

After participants reviewed each company's webpages they were asked to respond to a few filler questions about their attraction to each potential employer. After repeating this process for each company, and before presenting the manipulation check items, participants responded to two open-ended questions: “Do you think the information on the We Care page affected your desire to work at Active Style? Why or why not?” and “Does the information on the We Care page suggest anything to you about Active Style or what it would be like to work there?.” Participants were encouraged to take a moment to think about their responses and to write as much as they wished, and we emphasized that “this information is important to our study.” Participants then responded to two manipulation check items on a Likert-type scale from 1 (*Strongly Disagree*) to 7 (*Strongly Agree*): “Active Style tries to contribute positively to the communities in which it does business,” and “Active Style makes an effort to reduce its impact on the environment.”

## Results

### Content analysis protocol

The third author, who was blind to experimental condition, conducted an initial content analysis of written responses to the two open-ended questions about the “We Care” webpage using a grounded theory approach (Glasser and Strauss, 1967). Also while blind to experimental condition, the first author reviewed and refined the results of the first round of coding, and conducted all coding of a subset of cases pertinent to Research Question 3. We first developed an *a priori* coding protocol based on existing theory and research that led us to include specific categories pertaining to the three signals and associated mechanisms studied by Jones et al. (2014): organizational values and perceived value fit; the employer's reputation and prestige, and anticipated pride; and the employer's prosocial orientation and expected employee treatment. We also included coded categories based on speculation about potential signals from CSR with respect to the company's financial standing and compensation levels (Jones and Willness, 2013), the type of employees who work in the organization (Jones et al., 2014) and the overall work climate (Zhang and Gowan, 2012). During the coding process we added categories to code statements that reflected potentially new signal-based mechanisms that may affect job seekers' attraction to an employer known for its CSR (e.g., that employees who already work there share their employer's values), and recoded all previously coded responses for any references to the new category. We also coded for statements containing any explicit references to having negative reactions to the CI or ES information.

We adopted a stringent approach to coding the information provided by participants, such that all coding reported herein is based on coding written statements that contained *explicit* references that pertained to the subject of a given category, while excluding statements that only *implied* the subject of a given category. For example, a statement that we coded as an explicit reference to Active Style's specific values in relation to community involvement was, “They care about helping the community and volunteering”; a statement that we did not code due to its lack of explicitness was, “The We Care page did seem more community friendly.” We later note a single exception in which we coded statements that clearly suggested a sense of skepticism without explicitly using that term or a synonym.

### Manipulation checks

Analyses showed the manipulation of CI vs. ES information on the “We Care” page functioned as intended. Participants in the CI condition rated the CI manipulation check item significantly higher than in the ES condition: *t*_(106)_ = 4.24, *p* < 0.001 (*M* = 5.78, *SD* = 0.86 vs. *M* = 5.07, *SD* = 0.87). As expected, the reverse was true for ratings on the ES manipulation check item: *t*_(106)_ = −7.09, *p* < 0.001 (*M* = 4.56, *SD* = 1.02 vs. *M* = 5.93, *SD* = 0.99).

Our tacit assumption was that some study participants would become more attracted to the target employer after reading its “We Care” webpage containing either CI or ES content. To assess this assumption we analyzed their written responses to the first open-ended question: “Do you think the information on the We Care page affected your desire to work at Active Style? Why or why not?” In the CI condition, 35 participants (64.81%) explicitly claimed they were more attracted to the prospect of working for Active Style as a result of the CI information. One of these participants wrote, for example, “I definitely think the information on the ‘We Care’ page affected my desire to work at Active Style. I mean it is a great attribute to the company that they would do volunteer work.” A similar proportion of participants in the ES condition claimed the same (*n* = 34, 62.96%), as reflected in this comment: “The information portion in ‘We Care’ was one of the main reasons I rated the company above the other. It was important to me that they felt environmental action was important and they were taking steps to reduce their waste and consumption.” Thus, across both conditions, almost two-thirds of the participants claimed the information on the “We Care” page enhanced their attraction to the employer.

### Hypotheses testing

The results of the content analysis are shown in Table 1 with respect to Hypotheses 1 through 3 about the three signal-based mechanisms supported in Jones et al. (2014). Reported in Table 1 are frequency counts and percentages reflecting the number of participants in each condition whose responses to the two open-ended questions included one or more explicit reference(s) pertinent to each coded category. The results for each coded category are organized within the three signal-based mechanisms that are labeled in the table using italics. In reporting the results for each italicized mechanism, we only counted a participant once in the event that his or her responses were coded in multiple categories reported for that mechanism.

**Table 1:** **Frequencies and Percentages of Responses about an Employer's Community Involvement (CI) or Environmentally Sustainable (ES) Practices: Explicit Comments Pertaining to Three Signal-Based Mechanisms**.

**Coded categories within signal-based mechanisms**	**CI condition** ***n* = 54**	**ES condition** ***n* = 54**	**Representative quotes**
	**Frequency (%)**	**Frequency (%)**	
*Employer's Specific Values and Perceived Value Fit*	*27 (50.00%)*	*33 (61.11%)*	
Employer's CI- or ES-related values	25 (46.30%)	30 (55.56%)	#1.1: The information on the “We Care” page strongly affected my desire to work at Active Style. It appears that Active Style is not out just to maximize profits but to give back to the communities it is a part of.
			#1.2: It suggests that if they care about the environment and sustainable progress, then they probably are thoughtful about other transactions as well.
Participant's specific values relating to CI or ES	4 (7.41%)	5 (9.26%)	#1.3: The information on the “We Care” page increased my original desire to work at Active Style because I really believe in the Green Movement.
			#1.4: Personally, I would more likely work at a company that has similar values as I do and has ways of giving back to the community or supporting locals.
Fit between employer and personal values	4 (7.41%)	4 (7.41%)	#1.5: Because I am environmentally minded I think that the We Care page made me feel as though I would fit into the culture of the company.
			#1.6: I would more likely work at a company that has similar values as I do and has ways of giving back to the community.
*Employer's Prestige and Anticipated Pride*	*8 (14.81%)*	*1 (1.85%)*	
Pride as an employee	2 (3.70%)	–	#1.7: Yes, because people want to feel proud of where they work.
			#1.8: It is a place I would feel proud to work for. All their emphasis on philanthropy and the community is something I like to see in companies.
Employer respectability	2 (3.70%)	1 (1.85%)	#1.9: I realized that this organization cares about its employees. More importantly it cares about the surrounding community and strives to reach out. There connection with Clothes For Kids is very unique and the donation decision is very respectable.
			#1.10: It becomes a bonus if I found myself in a location and position that I greatly enjoyed and the company just so happened to be one that people greatly respected because of their policies toward the environment.
Employer focuses on its image and reputation	5 (9.26%)	–	#1.11: Suggests that the company as a whole cares deeply about its image in the community.
			#1.12: The page suggests that they want to be well respected in the community and want their employees to care about the community.
*Employer's Prosocial Orientation and Expected Treatment of Employees*	*27 (50.00%)*	*23 (42.59%)*	
Employer's prosocial orientation	24 (44.44%)	16 (29.63%)	#1.13: I think it gives me an idea that they care about being good morally as a company.
			#1.14: The “We Care” page, if anything, is evidence that the management cares about people.
Employer cares about more than profit	8 (14.81%)	6 (11.11%)	#1.15: It suggests that they really do care about more things than just gaining more profits.
			#1.16: It showed that Active Style cares about not only making a profit, but also about how it affects others.
Employer cares about and treats its employees well	16 (27.82%)	17 (31.48%)	#1.17: The “We Care” page is evidence that the management cares about people. Employees are people so that mentality would be the same inside the company.
			#1.18: I would think since Active Style cares so much about the environment and its externalities on others, they would treat their employees with the same respect.
Inferring favorable employee treatment from CI or ES information	6 (11.11%)	7 (12.96%)	#1.19: I got the impression that they care for the environment and social impact of the company, and companies like this typically take great care of employees.
			#1.20: If they care that much about the community they are a part of then they must care about their employees as well, making it a great place to work.

*Frequency counts and percentages reflect the number of participants in each experimental condition who wrote one or more explicit statements that we coded in each category based on responses to two open-ended questions (e.g., Question 1: “Do you think the information on the We Care page affected your desire to work at Active Style? Why or why not?”). Results in italics are reported for each of the three signal-based mechanisms studied by Jones et al. (2014), and reflect the cumulative frequency counts from the associated categories while counting any given participant only once*.

Included in all tables are two representative quotes taken from written statements coded within each category, where applicable. Each quote is numbered for ease of reference herein, with the first digit reflecting the table number followed by a decimal and a number that reflects its order in the table (e.g., quote “#1.1” refers to the first quote listed in Table 1). In the results below, we refer the reader to illustrative quotes listed in the tables using the corresponding reference numbers in parentheses, and in most cases these references do not reflect an exhaustive list of coded comments, as reflected in the frequency counts displayed in the tables.

Hypothesis 1 was that CI and ES information sends signals to job seekers about the employer's organizational values, from which they infer how well those values fit with their own values (i.e., perceived value fit). Table 1 shows that support was found for Hypothesis 1, such that 50% and 61% of participants in the CI and ES conditions, respectively, made explicit references pertaining to the perceived value fit mechanism. Most of those participants referred to the employer's specific values pertaining to CI or ES (#1.1, #1.2), and some people mentioned their own values pertaining to CI or ES (#1.3, #1.4). Providing the most direct support for this hypothesis, eight participants explicitly mentioned they were attracted by the fit between the employer's values and ethics with their own (#1.3, #1.4, #1.5, #1.6). For instance, a participant not quoted in Table 1 wrote, “Yes it affected my desire to work there. The information on that page allowed me to connect with the company. I felt like I knew something about their values and ethics, and because I have the same ethics I would want to work there more.”

Hypothesis 2 was that an employer's CI and ES sends signals about the employer's reputation and prestige, which informs job seekers' beliefs about how proud they would feel to be one of its employees. Table 1 shows that we found some, but relatively limited, support for Hypothesis 2. Two participants in the CI condition wrote explicit statements regarding feeling proud about working for the employer in question (#1.7). A participant quoted in Table 1 wrote, “It is a place I would feel proud to work for. All their emphasis on philanthropy and the community is something I like to see in companies” (#1.8). Three other participants wrote statements reflecting a belief that the target employer was “respectable” (#1.9), or the importance of other people respecting the employer because of its CI or ES practices (#1.10). Also relevant to this hypothesis, albeit not providing direct support for it, five participants noted the employer appears to care about its image and reputation (#1.11, #1.12).

Hypothesis 3 was that CI and ES send signals to job seekers about the employer's prosocial orientation, from which they infer how favorably the employer likely treats its employees. Table 1 shows that 50 and 43% of participants in the CI and ES conditions, respectively, made explicit statements reflecting signals about the employer's generalized prosocial orientation, their resulting inferences about expecting that the employer treats its employees well, or both. Most of those participants commented that the CI or ES information suggested that the employer was pro-socially motivated in a general sense (#1.13, #1.14), and a subset of them framed it as the employer cares about more than just generating profit (#1.15, #1.16). Providing support for Hypothesis 3, about 30% of the participants in each condition inferred from CI or ES that the employer likely treats its employees well (#1.17, #1.18). The link between signals about the employer's prosocial orientation and the resulting inference about favorable employee treatment was reflected in comments from seven participants in each condition (#1.17, #1.18, #1.19, #1.20), as illustrated in this comment from a participant not quoted in Table 1: “I got a sense that if they care for future generations they would also really care about their employees as well. It gave me a feeling that they would be more concerned for my well-being.”

### Assessment of research questions

Table 2 displays frequency counts, percentages, and representative quotes pertaining to Research Question 1 about evidence for other potential and previously untested mechanisms. Table 2 suggests that other potentially important signal-based mechanisms might exist. First, among 20 participants, an employer's CI and ES appeared to send signals that informed inferences about a positive work environment (#2.1, #2.2; this category did not include coded statements reported in Table 1 about the employer caring about and treating employees well). Other potential signals from CI and ES were suggested by comments about the employer's positive financial standing (#2.3, #2.4), opportunities within the company (#2.5), and the employer's future-oriented focus (#2.6, #2.7). Comments from 12 people suggested they inferred from CI and ES that the company's employees are cohesive and share similar values (#2.8, #2.9), as well as other characteristics of its employees and the potential to form friendships (#2.10, #2.11). For instance, a participant not quoted in Table 2 wrote, “It suggests that the employees are considerate people who care about the environment and others. They seem like they would be friendly people who are enjoyable to work with.”

**Table 2:** **Frequencies and Percentages of Responses about an Employer's Community Involvement (CI) or Environmentally Sustainable (ES) Practices: Evidence for other Signal-Based Mechanisms that Potentially Enhance Job Seeker Attraction**.

**Coded categories within signal-based mechanisms**	**CI condition** ***n* = 54**	**ES condition** ***n* = 54**	**Representative quotes**
	**Frequency (%)**	**Frequency (%)**	
Positive work environment	8 (14.81%)	12 (22.22%)	#2.1: I think it would be a good environment to work in.
			#2.2: It does seem to me like there might be more of an upbeat positive atmosphere working there that could be potently more optimistic about things in general.
Positive financial standing	2 (3.70%)	4 (7.41%)	#2.3: They're financially secure.
			#2.4: I think the more profitable businesses in the future will need to incorporate some form of ecological awareness into their operations. Any company that appears to have this direction would appeal to me simply because to me it would signal that they have long term success potential.
Opportunities within the company	1 (1.85%)	–	#2.5: The “We Care” page showed that their company offered more opportunities.
Company is adaptable, future oriented, or cutting edge	–	5 (9.26%)	#2.6: It made it seem like a very “up and coming” company with a lot of goals for the future.
			#2.7: Companies that are implementing ways of being environmentally friendly are steps ahead of others who have not yet done the same.
Employees are cohesive and share similar values	2 (3.70%)	3 (5.56%)	#2.8: Workers of this company are closer because they stand for same causes, and also because they made the choice to work for this company knowing what it was doing to make a difference.
			#2.9: Sustainability is something that many people either care about greatly or don't really think about. I think it would be almost guaranteed to have people with similar values and beliefs which would probably make for a happier and cohesive work environment.
Other employee characteristics and potential friendships	5 (9.26%)	7 (12.96%)	#2.10: Yes because it tells you about the people who currently work there and who you would be working with.
			#2.11: I feel that they are more of a tight knit group; people I could really get along with through working in groups.

*Frequency counts and percentages reflect the number of participants in each experimental condition who wrote one or more explicit statements that we coded in each category based on responses to two open-ended questions*.

Research Question 2 focused on potential differences between CI and ES in terms of the signals people receive and the inferences they draw from them. As seen in Table 1, no clear evidence was found for CI-ES differences with respect to the three hypothesized signal-based mechanisms, although a trend emerged for the anticipated pride mechanism, such that a greater number of relevant comments were made by people in the CI condition. The results in Table 2 about other possible signal-based mechanisms likewise show relatively little evidence for any CI-ES differences, with the possible exception of inferences about the company being adaptable and future-oriented for which all five comments came from the ES condition (#2.6, #2.7).

Research Question 3 was about whether and why some job seekers might be less attracted to an employer because of its CI or ES. In the CI condition, 17 people claimed the “We Care” page had no effect on their attraction (31.48%), and two people reported they were *less* attracted as a result of that information (3.70%). One of the latter two appeared to respond negatively due to a misunderstanding that his or her volunteering on behalf of the company would occur outside of the normal work hours on his or her free time (#3.15). The other participant who claimed to be less attracted due to the CI information wrote, “Yes, I thought it looked like it was just a load of BS to try and enhance their image” (#3.16). Results from the ES condition were remarkably similar to the CI condition: 19 participants reported that the employer's ES had no effect on their attraction (35.19%), and one participant claimed it made him or her less attracted (1.85%), writing, “It seems like the slogan is only there to attract prospective employees to apply and to attract potential customers into the target market” (#3.27). The answer to Research Question 3, then, is that only a small proportion of the 108 participants (*n* = 3, 2.78%) reported being less attracted to the employer because of its CI or ES, and the two participants who correctly interpreted that information expressed skepticism and cynicism about the employer's motives.

We conducted additional analyses to inform reasons why an employer's CSR might be ineffectual or counterproductive in enhancing job seeker attraction. We combined the 36 participants—exactly one-third of the sample—who reported that the CI or ES information had no effect on their attraction with the three people who claimed they became less attracted to create a subsample for these additional analyses (*n* = 19 in the CI condition, and *n* = 20 in the ES condition).

Table 3 displays frequency counts, percentages, and representative quotes pertaining to these analyses among this subsample (*n* = 39). Presented in the first section of the table under the “Signals Sent” heading are results based only on responses to the second open ended question about whether and what signals participants might have received from the CI or ES information. Results show that 13 people (33% of the subsample) claimed they received no signals about the work environment or the company more broadly (#3.1, #3.2), and three others (8%) described negative signals (#3.3, #3.4). Intriguingly, 23 people in the subsample (59%) claimed they received positive signals (including a few signals that were somewhere in between being positive and neutral in their tone), as illustrated in quotes #3.5 and #3.6. Despite receiving positive signals, 22 of these participants claimed their attraction was unaffected by the employer's CI or ES, and one other participant claimed to be less attracted as a result of the employer's CI practices.

**Table 3:** **Frequencies and Percentages of Responses about an Employer's Community Involvement (CI) or Environmentally Sustainable (ES) Practices among Participants who Reported No Effect (*n* = 36) or a Negative Effect (*n* = 3) on their Attraction**.

**Coded categories within signal-based mechanisms**	**CI condition** ***n* = 19**	**ES condition** ***n* = 20**	**Representative quotes**
	**Frequency (%)**	**Frequency (%)**	
**SIGNALS SENT**			
No signals	6 (31.58%)	7 (35.00%)	#3.1: I don't recall that page suggesting anything about what it would be like to work there.
			#3.2: Nothing in specific.
Negative signals	1 (5.26%)	2 (10.00%)	#3.3: It gives me the idea that the company is very concerned about their surroundings, which could potentially detract from their profits.
Work environment	1 (5.26%)	–	
Company and other	–	2 (10.00%)	#3.4: They seem to push employs to become involved in non-profits.
Positive or neutral signals	12 (63.15%)	11 (55.00%)	#3.5: It suggests that many employees would be similar to me in an outdoorsy way which would enhance my experience working for them.
Work environment	3 (15.79%)	8 (40.00%)	
Company and other	9 (47.37%)	3 (15.00%)	#3.6: An involved company in the community and one that doesn't just focus on manufacturing and profit.
**REASONS CI OR ES DID NOT ENHANCE ATTRACTION**			
Poor fit with employer	5 (26.32%)	3 (15.00%)	#3.7: I don't have much desire to work for a company like that.
			#3.8: I am interested in a particular field which Active Style is not in.
Compensation priority	1 (5.26%)	2 (10.00%)	#3.9: It didn't affect my desire in the least bit…When companies donate percentages of their revenue to certain organizations it doesn't make me want to work there more. All I care about is my personal pay and how well I like my job.
			#3.10: I guess if I was offered a job at both places that was identical in terms of pay and promotion opportunity then I would choose ActiveStyle, but other than that it's not as important to me.
Other priorities	3 (15.79%)	4 (20.00%)	#3.11: It would be far more important to me that I felt comfortable working there, liked my role there, and most importantly that I be compensated well.
			#3.12: It didn't affect my desire to work there. It's nice to see that a company cares about specific things, but when looking for a job, there are more important things that I would like to find out about the company before I find out what the company's values, ethics, etc.
Detracts from profits or company success	–	2 (10.00%)	#3.13: It gives me the idea that the company is very concerned about their surroundings, which could potentially detract from their profits.
			#3.14: It seems as though they are making many great steps toward helping the environment but a company cannot possibly be successful if they concentrate more on being socially responsible than their mission statement.
Misunderstood information	1 (5.26%)	–	#3.15: I would be asked to do community service in my free time in order to keep the companies vision of “we care” in mind. Therefore, more of my free time goes to being an employee at the company, making me not want to work there.
Skepticism and cynicism about CI or ES	7 (36.84%)	6 (30.0%)	#3.16: I thought it looked like it was just a load of BS to try and enhance their image.
			#3.17: It didn't really affect my desire too much because I feel like a lot of companies just say that they care and are environmentally friendly because it looks good to customers.
**FACTORS AFFECTING SKEPTICISM AND CYNICISM ABOUT CI OR ES**			
Must see or experience to believe	1 (5.26%)	2 (10.00%)	#3.18: No because you cannot know how the work environment is until you see the work place or actually start to work there.
			#3.19: I believe that every company is going to act like they care. I don't know the validity of the page because I haven't experienced the actual work environment.
More detail needed	2 (10.53%)	1 (5.00%)	#3.20: It gives no indication of how the “we care” aspect would be backed up and nothing about me specifically engaging in those activities.
			#3.21: It suggests that they care more than just sustaining profits. However, it was not detailed enough to prove anything really.
Prior experience	1 (5.26%)	–	#3.22: The company that I work for claims to be “paperless” and environmentally friendly, but my experiences tell me the exact opposite…I can't even begin to tell you about the vast amounts of waste that is produced. For me, pages like the “we care” page don't mean anything.
Poor fit with business	1 (5.26%)	–	#3.23: Their program seems like it is more being done for the sake of being done because other than the clothes drive it doesn't relate to their business or local community at all.
Non-distinctive impact	1 (5.26%)	2 (10.00%)	#3.24: It is nice to know that they care about the community, but there are many other companies out there that do much more than donate 2% of their revenue and have employee volunteering.
			#3.25: I believe that every company attempts to be environmentally friendly and sustainable and their proposal wasn't impressive enough to stand out.
Motives for CI or ES	3 (15.79%)	3 (15.00%)	#3.26: It suggests that the management is concerned with their effect on the environment. Whether they truly care or are just practicing good CSR/Triple bottom line theory for the purpose of improving profitability is anyone's guess.
			#3.27: It's a growing company that may or may not be successful. It seems like the slogan is only there to attract prospective employees to apply and to attract potential customers into the target market.
All firms claim to care	2 (10.53%)	3 (15.00%)	#3.28: I think every company would write that on their website.
			#3.29: I honestly didn't pay too much attention to the “we care” page because I feel that is a much more common thing to include on websites as many companies are going green and giving back. Because of this many of these statements don't seem as personal.

*Frequency counts and percentages reflect the number of participants in each experimental condition who wrote one or more explicit statements that we coded in each category based on responses to two open-ended questions, except for results under the “Signals Sent” heading that are based only on responses to Question 2: “Does the information on the We Care page suggest anything to you about Active Style or what it would be like to work there?”*.

To understand why a little more than one-third of the total sample claimed the employer's CI or ES did not enhance their attraction, or even detracted from it, we coded responses to both open-ended questions among the subsample described above, and the results are presented in Table 3 under the second heading (“Reasons CI or ES Did Not Enhance Attraction”). Eight people, or 20.5% of the subsample, described a general lack of fit with the advertised positions or the employer (e.g., #3.7, #3.8). Three others stated that compensation or pay was a more important consideration (#3.9, #3.10, #3.11), and all three were among the six participants who listed other priorities, such as how much they enjoyed their job role (#3.9, #3.11) and promotion opportunities (#3.10). A seventh person referred to unspecified priorities (#3.12). Two participants stated their belief that the employer's investments in ES might detract from its profits or success (#3.13, #3.14).

As shown in Table 3, the most prevalent theme among the stated reasons for not being attracted by CI or ES was a sense of skepticism and cynicism. Specifically, comments from 13 people—one-third of the subsample—did not include explicit references to skepticism, but nevertheless suggested they were skeptical and cynical about the employer's CI or ES practices. As stated by one individual who is not quoted in Table 3, “In a job I look for a company that cares about its employees and the environment, etc.; however, I feel like most companies say that. It would've been better had there been pictures with their employees so I could picture myself working there (doing something good).”

We further coded the responses from these 13 people and identified seven interrelated sources of skepticism and cynicism that are listed under the third heading of Table 3: “Factors Affecting Skepticism and Cynicism about CI or ES.” First, three people commented that they needed to see or experience the employer's CI or ES to believe it (#3.18, #3.19). Second, three individuals wrote comments suggesting that they would need more detail on the We Care page for them to draw meaningful inferences from the employer's CI or ES (#3.20, #3.21). A third apparent source of skepticism and cynicism was rooted in one participant's prior experience with an employer's greenwashing (#3.22). A fourth source was suggested by one person who appeared skeptical because he or she felt the CI practices were unrelated to the employer's business model (#3.23). Fifth, three people seemed to believe that the employer's positive social or environmental impact was too small or not distinctive enough to affect their attraction (#3.24, #3.25). A sixth source of skepticism and cynicism was expressed by six participants who questioned the nature of the motives they attributed to the employer's investments in CI or ES. For instance, participants questioned whether the employer's ES was motivated by genuine concern vs. the pursuit of profits (#3.26), by a desire to enhance their image (#3.16), or to attract customers (#3.17, #3.27). A seventh apparent source of skepticism and cynicism was communicated by five participants who asserted that many companies now claim to be socially or environmentally responsible (#3.28, #3.29).

## Discussion

We designed and conducted this study for two overall purposes. First, we tested hypotheses as part of a substantive replication to assess the generalizability of three signal-based mechanisms supported in recent research (Jones et al., 2014) while using an alternative study design, data type, and analytic approach. Our study deign allowed study participants to tell us the reasons they were attracted by an employer's CSR, rather than potentially priming them through survey items used to measure specific hypothesized mechanisms. Second, we sought to advance theory and research in this area by assessing research questions to uncover plausible yet previously unidentified signal-based mechanisms, explore possible differences between CI vs. ES in the underlying mechanisms involved, and illuminate potential reasons why some job seekers might be less attracted to an employer because of its CI or ES. Next we discuss the implications of our findings, study limitations, and implications for recruitment practice.

### Evidence for three previously established signal-based mechanisms

We found corroborating evidence for two mechanisms shown in previous research (Jones et al., 2014) to explain the effects of an organization's CSR on job seekers' attraction to a hiring company among approximately one-half to two-thirds of the sample with respect to an employer's CI or ES practices. Our content analysis of responses to open-ended questions suggested that information about these types of CSR practices sent signals about the organization's specific values that informed participants' inferences about perceived value fit, and signals about the employer's prosocial orientation that informed participants' inferences about the expected treatment of employees. We found some, but relatively less, supporting evidence for the anticipated pride mechanism, despite its emergence as a particularly strong mechanism in the Jones et al. (2014) studies. A possible explanation is the advertised positions were for entry level jobs, and the younger-aged participants were less inclined to consider these jobs as long-term career-oriented positions for which the employer's reputation would be relatively more important.

Another explanation for the modest support found for signals about an employer's prestige and resulting inferences about anticipated pride is that these psychological processes operate outside of people's conscious awareness. If so, support for this mechanism may only be found when people are primed to consider it through the completion of survey items about anticipated pride or organizational prestige, as in Study 1 and Study 2 of Jones et al. (2014), respectively. Consistent with this possibility, participants in Jones et al.'s Study 1 reviewed webpage content for Active Style and for two other employers that was very similar to the materials we used in the current study. *After* those participants completed items about anticipated pride and other measures for all three employers, they were asked to rank order and justify their top employment option. Among the majority of participants who ranked the target company as their top choice, 11 and 14% of participants in the CI and ES conditions, respectively, made references to the employer's prestige and reputation, in contrast to the relative lack of similar references found in the present study. The same patterns of results, however, also suggest another possibility—the employer prestige-anticipated pride mechanism may not explain the CSR-attraction relationship at all, and the prior evidence found in the Jones et al. studies, as well as in Behrend et al. (2009), could reflect an artifact of people being primed to respond accordingly when asked to complete the associated measures. Although we think the first of these two possibilities is the more likely explanation, further research is needed to investigate the validity of the employer prestige and anticipate pride signal-based mechanism. Given Willness and Jones's (2013) assertion that the source of CSR information affects how people perceive it, we suspect that this signal-based mechanism would be stronger when an employer's webpages include explicit references to third-party endorsements and honors received for its CI or ES practices.

### Evidence for other signal-based mechanisms and CI vs. ES differences

We explored whether other previously unstudied signal-based mechanisms might hold promise for extending the understanding of why job seekers tend to be attracted by CSR. Our findings suggest that some job seekers may be attracted to employers known for their CI and ES due to signals that lead them to make inferences about the characteristics and shared values of the employees who work there, the potential to form new friendships, the employer's positive financial standing, opportunities for employees within the company, and the employer's adaptability and future orientation. The most common signal-based inference in our data, aside from those relating to the three hypothesized mechanisms, was about the likely existence of a positive work environment.

These findings highlight potential signal-based mechanisms that should be tested in future studies to ultimately provide a basis to inform and develop a signal-based theory of CSR and recruitment. Scholars have observed that recruitment researchers who draw on signaling theory usually do not directly test, or even delineate, the precise signals job seekers are proposed to receive from a given information source, and rarely do researchers describe how those signals are linked to the inferences job seekers are proposed to draw from them (Celani and Singh, 2010). We speculate that the inferences observed in this study about the employer's positive work environment and opportunities for employees are likely based on signals from CSR about the employer's prosocial orientation, and that inferences about employee characteristics most likely follow from signals about the employer's specific values pertaining to their CSR practices. We further speculate that inferences about the employer's future orientation are based on signals from CSR about the employer's focus on multiple stakeholders, and that inferences about its financial stability are based on signals about the employer's presumably discretionary expenditures to support these CSR practices.

We also explored potential differences in the signal-based mechanisms associated with an employer's CI vs. ES practices, and we did not find clear evidence for any differences. Two trends emerged in these data, however, that we believe are worthy of future investigation. First, consistent with Jones et al.'s (2014) speculation, people in the CI condition made four comments about anticipated pride and the employer's respectability vs. only one comment in the ES condition. Although the low number of comments prohibits us from inferring that a CI-ES difference on the anticipated pride mechanism truly exists outside these data, we think this difference is quite plausible and it should be explored in future research. Second, all five comments that were coded as inferences about the company being adaptable and future-oriented came from the ES condition. This possible difference should be explored in future research, especially given that it is a logical leap to make this inference based on a firm's investments in environmental sustainability in the context of recent media attention and global action to combat the effects of climate change.

### Study limitations

A potential limitation of our study was its reliance on younger-aged student participants who may hold more favorable attitudes toward CSR compared to the larger job seeker population. While this may limit the generalizability of our results, our younger sample reflects an important demographic group that represents a large proportion of new labor market entrants. Indeed, many employers actively tailor their recruitment messages toward student-aged populations (Dineen and Noe, 2009). Notwithstanding the practical relevance of our findings to recruiting organizations, we repeat a call to conduct CSR-recruitment studies using diverse samples, given that the majority of extant research has used student-based samples as we did (Jones and Willness, 2013).

Given our focus on analyzing written responses to open-ended questions, another possible limitation is that the proportion of participants who claimed to be more attracted by the CSR information is inflated due to socially desirable responding. Notably, however, the methodology ensured complete anonymity and confidentiality, which we highlighted in the instructions. Moreover, the support we found for two signal-based mechanisms is consistent with theory and findings based on quantitative data as we have described. Our data is also limited by the capacity of individuals' self-insights, and their motivation and ability to accurately translate their meta-cognitions to written responses.

### When job seekers are unaffected or repelled by CSR: an agenda for future research

To our knowledge, this study represents the first examination in the CSR-recruitment literature that addresses questions about whether and why CSR might “go wrong.” We believe this is a particularly important topic, both theoretically and practically, for future inquiry. Common sense suggests that not every job seeker will respond positively to an employer's CI or ES, given variability in people's values and beliefs pertaining to such practices. However, our findings point to other, more nuanced, reasons why one-third of our sample reported that their attraction was unaffected by the employer's CI or ES, and a few others reported being *less* attracted as a result of those practices.

Some of the stated reasons for their lack of CSR-based attraction are somewhat predictable, as some participants described a general lack of fit, or other priorities, such as their compensation or the nature of the job role. Specific to the discretionary nature of many CSR investments, however, were concerns raised by two people about the employer's ES potentially detracting from its profits or success. Researchers should study the contexts in which such an inference is more or less likely, such as the effects of an employer explicitly stating that the firm's ES practices are central to its strategy and revenue generation.

Other statements reflected a common theme among people who claimed they were not attracted by the employer's CSR: they were skeptical and cynical about its CI and ES claims. Consistent with Willness and Jones's (2013) suggestion that job seekers might react negatively when they question the credibility of CSR claims or suspect greenwashing, one-third of those who claimed they were not more attracted by the employer's CSR made statements that pointed to seven plausible inter-related sources of skepticism and cynicism. We believe this to be a particularly fertile area for future research that has considerable potential to advance theory and inform recruitment practice.

Our results suggest that some people were skeptical and cynical about CSR because they witnessed greenwashing in the past, or were reluctant to give much credence to the employer's claims without having witnessed or experienced the CI or ES practices themselves. A third apparent source of skepticism was communicated by people who claimed they needed more detail about these practices before they could draw meaningful inferences from them. Researchers should seek to identify factors that amplify or mitigate these apparent sources of skepticism and cynicism, and statements made by some non-skeptics suggest a few plausible factors for researchers to consider. One participant's comments point to employee testimonials as a potential remedy to skepticism and cynicism, writing “I think reviews by former employees or current employees is the best ways to learn about working there.” Statements from others highlight the potential value of communicating what Du et al. (2010) call *CSR Commitment* (i.e., tangible investments in CSR practices) and *CSR Impact* (i.e., quantifiable indicators of the social and environmental impact of those investments). For instance, one non-skeptic wrote, “The ‘We Care’ page was clearly laid out to emphasize important parts of their role in society. It didn't just say ‘we are involved’ or anything along those lines. It actually talked about it, and let the reader know the logistics.” Another non-skeptic wrote, “It shows that the company's name actually reflects its true feeling about the environment and is not just used as a marketing slogan. Also, showing specific information about “how” they care is appealing to me.” Although the information provided on the We Care page about CSR commitment and CSR impact was insufficient to override skepticism and cynicism among some participants, it did appear to be sufficient for others, such as one participant who wrote, “While it's possible the company could be over-exaggerating their claims, the page makes them seem genuinely friendly and considerate. I think it would be a good environment to work in.” In addition to advancing theory, studies that delineate the effects of employee testimonials, and indicators of CSR commitment and impact hold promise for providing tangible guidance to hiring companies and their recruiters.

Du et al. (2010) also emphasize the importance of managing stakeholders' attributed motives for a company's investments in CSR. Consistent with this assertion, several participants made comments suggesting that their beliefs about the employer's motives for investing in CI or ES represented another apparent source of skepticism and cynicism. These participants attributed self-interested motives to the employer, suggesting it only engaged in CSR to attract customers and employees, or as part of its broader pursuit of profit. Willness and Jones (2013) asserted that job seekers' skepticism about CSR claims might be reduced through recruitment messages and information that demonstrates the benevolent intentions and values-based motives behind the employer's CSR practices. This suggestion is supported by research on attributed motives for CSR, and so too is the notion that stakeholder respond positively to transparent messages in which firms communicate their longer-term strategic motives and efforts to manager stakeholder relationships through CSR (see Du et al., 2010). Doing the latter may help to combat a fifth apparent source of skepticism and cynicism observed in this study: a perceived lack of connection between the employer's CSR practices and its core business model (i.e., *CSR Fit*, Du et al., 2010).

Other seemingly skeptical and cynical participants described the employer's positive social or environmental impact as being too small and not distinctive enough to warrant their attention. Researchers should assess how the extent and distinctiveness of an employer's social and environmental impact might influence job seekers' skepticism and cynicism about its practices, and in turn, their attraction to the employer. A related seventh source of skepticism and cynicism observed in this study was reflected in several comments in which the employer was characterized as being no different than the majority of companies that now claim to be socially or environmentally responsible. This framing of the employer's CSR as a public relations ploy was implicitly echoed by a non-skeptical participant who suggested the employer's ES communicated little else to them beyond its environmental values given the information source, writing, “It doesn't really tell much about the morals and feelings of the company as a whole, rather than what one department has written about their philanthropy.” This latter comment is consistent with speculation by Willness and Jones (2013) who opined that negative reactions to CSR are more likely to occur when job seekers learn about the employer's practice through media it owns and controls, such as its website, but this assertion remains an untested empirical question.

Taken together, our findings about people's apparent skepticism and cynicism highlight a number of questions for future research. We enthusiastically repeat a call for more research on negative reactions to CSR, and on skepticism and cynicism more specifically (Willness and Jones, 2013), which we believe hold great promise for advancing theory and informing practice in this area.

### Other implications for recruitment practice

For recruitment practice, our results suggest that the net effect of leveraging CSR practices in employee recruitment is clearly a positive one from the perspective of a hiring organization. The majority of our participants—about two-thirds of them—reported they were more attracted to the employer as a result of its CI or ES, and we believe that understanding the underlying mechanisms involved can provide tangible guidance for recruitment practice.

Recruitment messages can be designed to highlight signals and the resulting inferences job seekers draw from them with regard to organizational values and inferences about perceived value fit (e.g., “We strive to grow our profits like any other business, but we believe we can do so while being a responsible member of our community, reducing our impact on the natural environment, and treating our employees and customers with the respect they deserve”). Recruitment messages can also highlight the prosocial orientation-expected treatment mechanism (e.g., “Just like we care about the people in our community, we care about the people who work here—we strive to set the gold standard for how employers should treat their people”). And, to the extent that the employer prestige and anticipated pride mechanism matters, recruiters can leverage that mechanism, too (e.g., “Our employees are proud of our sustainability efforts and we take time to celebrate the awards and honors we receive for these practices”). In sum, organizations' CSR practices communicate more than the practices themselves; our study suggests that, as a result of CSR, job seekers make a variety of inferences about an employer and its internal working conditions, and recruitment professionals have opportunity to leverage CSR practices to enhance applicant attraction and improve an organization's ability to identify and hire high performing employees.

## Ethics statement

The study design and processes used to protect the interests and rights of the human subjects involved in this study were approved by the Institutional Review Board at The University of Vermont, Committee on Human Research. All subjects provided their informed consent as per the approved protocol for this study.

## Author contributions

DJ contributed via conceptualization, writing, overseeing data collection, coding the data, and analyses. CW contributed via conceptualization and writing. KH contributed via conceptualization, data collection, and coding the data.

## Funding

This research was funded in part by the Nicole Maria Stata Summer Research Award awarded to the first author, and a Social Sciences and Humanities Research Council of Canada Standard Research Grant (# 410-2010-2100) awarded to the second author with the first author listed as Collaborator.

### Conflict of interest statement

The authors declare that the research was conducted in the absence of any commercial or financial relationships that could be construed as a potential conflict of interest.

## Appendix: webpage content about an employer's community involvement or environmentally sustainable practices

### Community involvement condition

At Active Style, we are committed to contributing to the communities we touch. We pride ourselves on being an industry leader with a number of cutting edge programs designed to contribute to our communities. When our customers buy Active Style clothing and apparel, they are not just wearing great clothes—they're wearing clothes that reflect our shared values about supporting our community.

#### Community philanthropy

We believe that business should be about more than just making money—we believe it is our responsibility to consider our impact on our communities in all the decisions we make. Since 2001, we've donated 2% of our annual after tax revenues to non-profit organizations, such as the United Way and local food banks.

#### Employee volunteering

Through our *ActiveVolunteer*^*TM*^ program, we help organize our employees to volunteer in non-profit organizations. Our employees serve various nonprofits, such as Habitat for Humanity and AIDS Walk. For three years running, the percentage of employees who volunteer through the program has increased by 10–13%. Based on the recommendations of a 2008 employee task force, we started a Clothes for Kids program through which we match each article of clothing our employees donate by donating a comparable article of Active Style clothing.

### Environmentally sustainable practices condition

At Active Style, we are committed to our environmental sustainability principles. We pride ourselves on being an industry leader with a number of cutting edge environmentally-friendly practices and programs. When our customers buy Active Style clothing and apparel, they are not just wearing great clothes—they're wearing clothes that reflect our shared values about protecting our environment.

#### Eco“logical” philanthropy

We believe that business should be about more than just making money—we believe it is our responsibility to promote environmental awareness and to consider our impact on the environment in all the decisions we make. Since 2001, we've donated 2% of our annual after tax revenues to eco-friendly organizations, such as the Sierra Club and Care2.

#### Employee-driven sustainability

Through our *EcoAction*^*TM*^ program our employees lead the way by creating and implementing creative programs, which have resulted in an 11% reduction of non-recycling waste company-wide in financial year 2010. For three years running, we have reduced our energy consumption by 10–13%. In 2008, we implemented three recommendations developed by our employee task force on eco-protection. We now use only recycled paper throughout the company, all meeting rooms have been converted to be “paperless,” and all offices participate in “energy-free” weekends where we close the offices and turn off and unplug all non-essential computers and equipment.
